# The contribution of δ subunit-containing GABA_A_ receptors to phasic and tonic conductance changes in cerebellum, thalamus and neocortex

**DOI:** 10.3389/fncir.2013.00203

**Published:** 2013-12-23

**Authors:** Zhiwen Ye, Thomas P. McGee, Catriona M. Houston, Stephen G. Brickley

**Affiliations:** ^1^Biophysics Section, Department of Life Sciences, Imperial College LondonLondon, UK; ^2^Department of Neuroscience, Physiology and Pharmacology, University College LondonLondon, UK

**Keywords:** GABA_A_ receptors, GABA_A_ receptor agonists, tonic inhibition, phasic inhibition, patch-clamp techniques

## Abstract

We have made use of the δ subunit-selective allosteric modulator DS2 (4-chloro-N-[2-(2-thienyl)imidazo[1,2-a]pyridine-3-yl benzamide) to assay the contribution of δ-GABA_A_Rs to tonic and phasic conductance changes in the cerebellum, thalamus and neocortex. In cerebellar granule cells, an enhancement of the tonic conductance was observed for DS2 and the orthosteric agonist THIP (4,5,6,7-tetrahydroisoxazolo[5,4-c]pyridin-3-ol). As expected, DS2 did not alter the properties of GABA_A_ receptor-mediated inhibitory postsynaptic synaptic conductances (IPSCs) supporting a purely extrasynaptic role for δ-GABA_A_Rs in cerebellar granule cells. DS2 also enhanced the tonic conductance recorded from thalamic relay neurons of the visual thalamus with no alteration in IPSC properties. However, in addition to enhancing the tonic conductance DS2 also slowed the decay of IPSCs recorded from layer II/III neocortical neurons. A slowing of the IPSC decay also occurred in the presence of the voltage-gated sodium channel blocker TTX. Moreover, under conditions of reduced GABA release the ability of DS2 to enhance the tonic conductance was attenuated. These results indicate that δ-GABA_A_Rs can be activated following vesicular GABA release onto neocortical neurons and that the actions of DS2 on the tonic conductance may be influenced by the ambient GABA levels present in particular brain regions.

## Introduction

GABA_A_ receptor heterogeneity influences the excitability of specific neuronal populations and is a major determinant of the drug sensitivity of different brain regions. For example, the discovery of the δ-GABA_A_R (Shivers et al., 1989; Wisden et al., 1992) led to the view that low ambient GABA levels in the extracellular space (Kekesi et al., 1997; Wu et al., 2007) was capable of generating a novel form of tonic inhibition in brain regions that expressed δ-GABA_A_Rs (Kaneda et al., 1995; Salin and Prince, 1996; Brickley et al., 2001; Stell et al., 2003; Drasbek and Jensen, 2006; Bright et al., 2007; Ade et al., 2008; Kirmse et al., 2008; Santhakumar et al., 2010). This particular member of the GABA_A_ receptor family has attracted clinical interest because the δ-GABA_A_R orthosteric agonist THIP has been shown to promote the slow thalamocortical oscillations associated with sleep (Faulhaber et al., 1997; Cope et al., 2005; Winsky-Sommerer et al., 2007). Gene knock-out studies have further demonstrated that δ-GABA_A_Rs are also an important target for the neurosteroid anesthetic alphaxalone (Mihalek et al., 1999; Spigelman et al., 2003). The motivation for pharmacologically targeting these δ-GABA_A_R populations (Brickley and Mody, 2012) has been further driven by a number of studies that have associated disruptions in δ-GABA_A_R activation with mood disorders (Smith et al., 1998; Maguire et al., 2005; Maguire and Mody, 2008; Sequeira et al., 2009; Feng et al., 2010), schizophrenia (Maldonado-Aviles et al., 2009), and epilepsy (Spigelman et al., 2002; Cope et al., 2009; Macdonald et al., 2010).

However, the high GABA affinity of δ-GABA_A_Rs (Bright et al., 2011) is such that a low-affinity orthosteric agonist such as THIP will be displaced from the GABA binding site at low GABA concentrations (Houston et al., 2012). Therefore, allosteric modulation of δ-GABA_A_Rs by neurosteroids (Mihalek et al., 1999; Belelli and Herd, 2003; Spigelman et al., 2003; Stell et al., 2003; Cope et al., 2005) represents a more effective strategy for enhancing the tonic conductance when ambient GABA levels *in vivo* are high (Houston et al., 2012). DS2 is an exogenous positive allosteric modulator that appears to act at a unique binding site on the δ-GABA_A_R (Wafford et al., 2009; Jensen et al., 2013). Unfortunately, DS2 has little utility from a clinical perspective due to a poor brain/plasma profile (Jensen et al., 2013), but DS2 could still prove useful in characterizing the contribution of δ-GABA_A_Rs to tonic and phasic responses. We are particularly interested in exploring the ability of DS2 to distinguish the contribution of δ-GABA_A_R to phasic and tonic conductance changes. For example, in cerebellar granule cells, the clear extrasynaptic location of δ-GABA_A_Rs (Nusser et al., 1995, 1998) has led to the hypothesis that these high-affinity receptors have a role that is both anatomically and functionally distinct from the lower-affinity synaptic GABA_A_ receptors. Consistent with this view, no difference in the kinetics of the inhibitory postsynaptic synaptic conductances (IPSCs) was reported in δ subunit knockout mice (Bright et al., 2011). However, it has previously been suggested that GABA spillover from the synaptic cleft may transiently activate extrasynaptic δ-GABA_A_Rs to generate a slow component to the IPSC decay (Rossi and Hamann, 1998; Hamann et al., 2002). We have chosen to re-address the involvement of δ-GABA_A_Rs to tonic and phasic conductance changes in a variety of different cell types of the cerebellum, thalamus and neocortex. The positive allosteric modulator DS2 was chosen as the action of this particular drug should only be apparent when δ-GABA_A_Rs are occupied by GABA. Therefore, this drug has the potential to assay the involvement of functionally relevant δ-GABA_A_Rs in the generation of tonic and phasic conductance changes.

## Materials and methods

### Acute slice preparations

Mice were routinely handled to reduce stress levels and brain slices were then prepared from adult (3–6 months postnatal) C57Bl/6J mice that were killed by cervical dislocation (in accordance with UK Home Office guidelines). The brain was rapidly removed and immersed in ice cold slicing solution. For cerebellar slices the slicing ACSF contained in mM; KCl 2.5, CaCl_2_ 1, MgCl 5, NaH_2_PO_4_ 1.25, NaHCO_3_ 26, glucose 11, glycerol 250. For thalamic and cortical slices the slicing solution contained (in mM: NaCl 125, KCl 2.5, CaCl_2_ 2, MgCl 2, NaH_2_PO_4_ 1.25, NaHCO_3_ 26, glucose 11, 1 kynurenic acid) pH 7.4 when bubbled with 95% O_2_/5% CO_2_. Slices were cut using a vibratome tissue slicer (Campden instruments) at a thickness of 250 μm and immediately transferred to a holding chamber containing slicing ACSF continuously bubbled with 95% O_2_/5% CO_2_. Once slicing was complete the holding chamber was transferred to a 37°C heat block for 40 min after which the slicing ACSF was gradually exchanged for recording ACSF (in mM: NaCl 125, KCl 2.5, CaCl_2_ 2, MgCl 2, NaH_2_PO_4_ 1.25, NaHCO_3_ 26, glucose 11, pH 7.4 when bubbled with 95% O_2_/5% CO_2_) and allowed to reach room temperature whilst the solutions were exchanged prior to electrophysiological recording experiments.

### Electrophysiology

Slices were visualized using a fixed-stage upright microscope (BX51W1, Olympus) fitted with a high numerical aperture water-immersion objective and a digital camera. The recording chamber was continuously perfused with the appropriate external solution. Solution entered the bath via a gravity perfusion system at a rate of 3 ml/min. Patch pipettes were fabricated from thick-walled borosilicate glass capillaries (1.5 mm o.d., 0.86 mm i.d., Harvard Apparatus) using a two-step vertical puller (Narishige, PC-10). Pipette resistances were typically less than 8 MΩ (for granule cells) and 3–4 MΩ (thalamic and cortical pyramidal cells) when back filled with internal solution. The internal solution contained (in mM) CsCl 140, NaCl 4, CaCl_2_ 0.5, HEPES 10, EGTA 5, Mg-ATP 2; the pH was adjusted to 7.3 with CsOH. The identity of each cell type was determined from location within the slice preparation and characteristic cell capacitance and input resistance. Biocytin (1.5 mg/ml) was included in the pipette solution so that neuronal cell-type could be confirmed from morphological features. For example, layer II/III pyramidal cells were first identified from their position in the neocortex and their characteristic cell capacitance of 125.3 ± 10.3 pF and input resistance of 214.7 ± 29.9 MΩ (*n* = 13). Location was later confirmed by comparison with Nissl staining of neocortical cell body layers and pyramidal cell morphology was unambiguously established in 7 out of the 13 recorded cells (data not shown). The amplifier headstage was connected to an Axopatch 200B or 700B amplifier (Molecular Devices; Foster City, CA). Fine and course movement of the pipettes were controlled by micromanipulators (PatchStar, Scientifica) mounted upon a fixed platform. The amplifier current output was filtered at 10 kHz (−3 dB, 8-pole low-pass Bessel) and digitized at 20 kHz using a National Instruments digitization board (NI-DAQmx, PCI-6052E; National Instruments, Austin, Texas). Data acquisition was performed using WINWCP (Version 4.1.2) and WINEDR (Version 3.0.9) kindly provided by John Dempster (John Dempster; University of Strathclyde, UK).

### Drug application

Direct drug application close to cerebellar neurons was performed with a gravity fed system via an eight unit solenoid driver controlled via TTL pulses produced from the WinEDR software. Up to eight different solutions flowed into a small diameter manifold tip placed close to the cell using a manual micromanipulator. For thalamic and cortical neurons bath application of drugs also used a gravity perfusion system with a flow rate of 2–3 ml/min into a total bath volume of ~2 ml. Solutions were transferred to the bath via PTFE tubing and were removed from the bath via a suction line connected to a peristaltic pump (Pharmacia Biotech P-1). 4-chloro-N-[2-(2-thienyl)imidazo[1,2-a]pyridine-3-yl benzamide (DS2, Tocris Bioscience, Bristol, UK) stock solutions were prepared so that the final DMSO concentration was less than 0.1% (Wafford et al., 2009). 4,5,6,7-tetrahydroisoxazolo[5,4-c]pyridin-3-ol hydrochloride (THIP, Sigma-Aldrich), Terodotoxin (TTX, Ascent Scientific) SR-95531 (gabazine—Ascent Scientific) and Kynurenic acid (Sigma-Aldrich) were prepared at the appropriate concentration in recording ACSF.

### Data analysis

In order to calculate the tonic conductance, all-point histograms of the current record were constructed and differences in the peak amplitude measured from a single Gaussian fit were used to calculate the amplitude of the standing inward current before and after DS2 (10 μM) application and also in the presence of 50–100 μM gabazine. Synaptic events were analyzed using WinEDR/WinWCP. Event detection was performed using amplitude threshold crossing or scaled template matching. Events were aligned on their initial rising phases and averaged synaptic waveforms were constructed from IPSCs that exhibited monotonic rises and an uninterrupted decay phase. Average baseline current levels were calculated during a 10 ms epoch immediately before each detected event and the peak amplitude was determined relative to this value. The decay of individual IPSCs was calculated as the charge transfer during the baseline corrected IPSC divided by the IPSC peak amplitude. This allowed a fit-independent estimate of the decay that could be determined for individual or averaged IPSCs.

### Statistical analysis

Distributions were compared using Origin 8.5 (Microcal, Century City, CA) using unconstrained least-squared fitting procedures, and statistical tests were performed at a 95% confidence level. Values stated are the mean ± the standard error of the mean (SEM).

## Results

### Cerebellar granule cells

**Figure 1:**
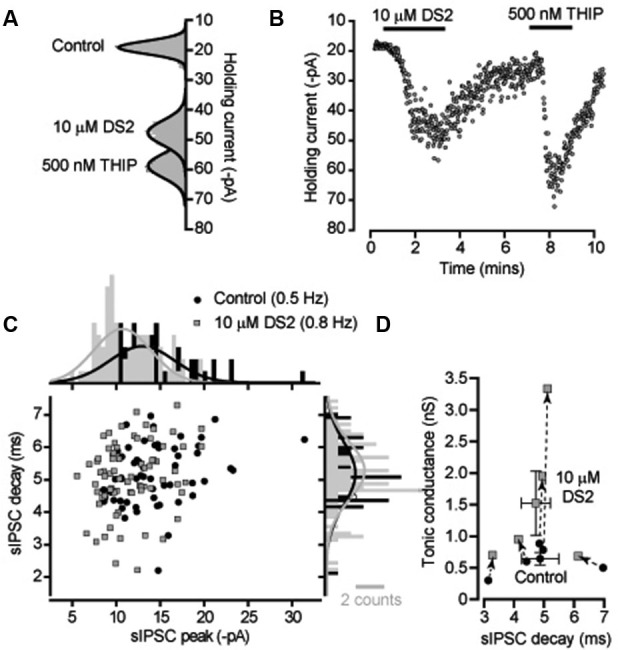
**DS2 application to cerebellar granule cells enhances the tonic conductance but does not alter IPSC properties**. **(A)** All-point histograms were constructed from 100 ms current traces (V_*H*_ −60 mV) recorded from a cerebellar granule cell in control conditions and in the presence of 10 μM DS2 and 500 nM THIP and the solid lines are Gaussian fits to the distributions. **(B)** The time course plot illustrates the change in average holding current calculated every 1 s from consecutive 100 ms current epoch. An enhancement of the tonic conductance was observed during DS2 and THIP applications. **(C)** Scatter plot of peak amplitude against decay time for all IPSCs recorded from this granule cell in the presence (filled squares, grey) and absence (filled circles, black) of DS2. The histograms on each axis illustrate Gaussian fits to the distributions constructed in the presence and absence of DS2. **(D)** Scatter plot of the average tonic conductance and average sIPSC decay before and after DS2 application. The data for individual cells are linked by dashed lines and arrows to illustrate the consistent increase in the tonic conductance that takes place with no consistent change in the IPSC decay. Superimposed on this plot are the average values across all cells (mean ± SEM, *n* = 5).

DS2 is expected to enhance the tonic conductance generated by the persistent activation of high-affinity δ-GABA_A_Rs with little action on IPSCs mediated by conventional synaptic γ-GABA_A_Rs. We initially compared the actions of DS2 with the more commonly used δ-GABA_A_R selective orthosteric agonist, THIP. Changes in tonic and phasic conductance were examined in adult cerebellar granule cells recorded from acute slice preparations. Gaussian fits to all-point histograms constructed from 100 ms current epochs were used to examine the time course of DS2 and THIP actions on the holding current (Figures 1A, B). From these data the tonic conductance was estimated to be 596 ± 84 pS (*n* = 7) in control conditions compared to 1631 ± 360 pS (*n* = 7) in the presence of 10 μM DS2 and 1658 ± 333 pS (*n* = 6) in the presence of 500 nM THIP. Therefore, the average tonic conductance recorded in the presence of DS2 and THIP was not significantly different (unpaired two sample *T*-test or Mann-Whitney *U*-test). The scatter plot of IPSC peak and decay shown for a single cerebellar granule cell in Figure 1C and the corresponding all-point histograms constructed from these distributions illustrates the similarity of the sIPSCs recorded in the presence and absence of DS2. In 5 cells the average frequency of sIPSCs was 0.8 ± 0.3 Hz in control versus 1.0 ± 0.3 Hz in DS2. The average sIPSC peak amplitude was 292 ± 50 pS in control versus 275 ± 36 pS in DS2 and the average decay of sIPSCs was 4.9 ± 0.6 ms in control versus 4.7 ± 0.5 ms in DS2. Therefore, as shown in Figure 1D, no significant change (paired sample *t*-tests) in sIPSC properties was observed in the presence of DS2 although a clear enhancement of the tonic conductance was observed using this allosteric modulator.

### Cerebellar interneurons

Cerebellar interneurons within the internal granule cell layer (Golgi cells) do not express high-affinity extrasynaptic δ-GABA_A_Rs and therefore do not exhibit any tonic conductance (Houston et al., 2012). As expected, no discernible resting tonic conductance could be measured in these cells and on average there was no significant tonic conductance associated with the application of 500 nM THIP (109 ± 69 pS, *n* = 6) or 10 μM DS2 (184 ± 107 pS, *n* = 6). The average sIPSC frequency was 9.8 ± 2.1 Hz in control versus 8.9 ± 1.9 Hz in DS2 (*n* = 6). The average sIPSC amplitude was 811 ± 231 pS in control versus 862 ± 269 pS in DS2. The average decay of sIPSCs was 8.3 ± 0.9 ms in control versus 8.5 ± 0.9 ms in DS2. Once again none of these changes were significant (paired sample *t*-tests). Therefore, it is clear that DS2, at the dose used in this study, does not influence conventional γ subunit-containing synaptic GABA_A_ receptors of the cerebellum.

### Thalamic relay neurons of the dLGN

**Figure 2:**
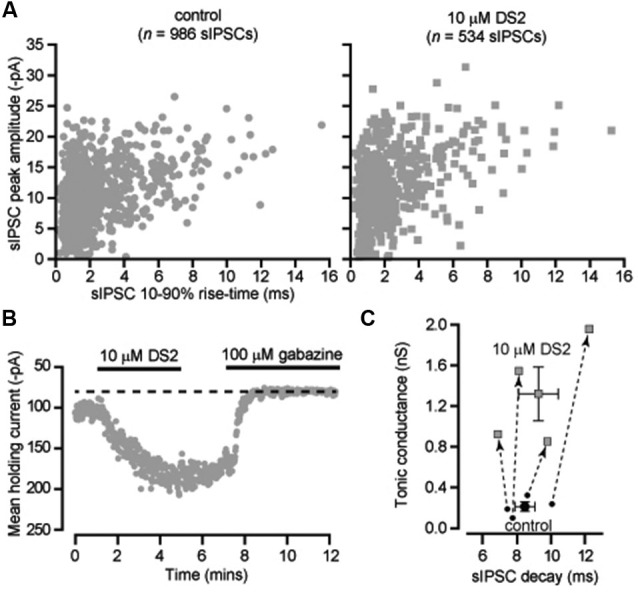
**DS2 enhances the tonic conductance recorded from dLGN thalamic relay neurons but does not alter IPSC properties**. **(A)** Scatter-plots of individual IPSC peak amplitude (pA) and 10–90% rise-time (ms) recorded from a dLGN thalamic neuron (V_*H*_ −70 mV) in the absence (control) and presence of 10 μM DS2. **(B)** Time course of the average holding current calculated every 1 s from consecutive 100 ms current epochs during the application of 10 μM DS2 and 100 μM gabazine. **(C)** Scatter plot of the average tonic conductance and average sIPSC decay before and after DS2 application. The data for individual cells are linked by dashed lines and arrows to illustrate the consistent increase in the tonic conductance that took place with no consistent change in the estimated IPSC decay. Superimposed on this plot are the average values across all cells (mean ± SEM, *n* = 4).

The ability of DS2 to selectively modulate the tonic conductance with little action on phasic inhibition has previously been reported for thalamic relay neurons recorded from the ventrobasal thalamus (Wafford et al., 2009). However, it is well known that in rodents the visual thalamus (dLGN) is the only thalamic relay nucleus to contain GABAergic interneurons (Ohara et al., 1983) and, therefore, it was important to establish whether DS2 actions would be similar in these two nuclei. Also, in our previous studies (Bright et al., 2007) we have demonstrated the presence of slow rising and slow decaying IPSCs in X-type relay neurons of the dLGN. Therefore, we first examined the actions of DS2 on fast and slow IPSCs recorded from dLGN thalamic relay neurons. As shown in Figure 2A, 10 μM DS2 did not influence the distribution of fast or slow IPSCs recorded from a dLGN relay neuron. Indeed, the average decay of sIPSCs was unchanged with a decay of 8.4 ± 0.6 ms in control conditions vs. 9.2 ± 1.2 ms in DS2. The average sIPSC amplitude was also 743 ± 137 pS in control vs. 689 ± 137 pS in DS2. The average sIPSC frequency was 8.6 ± 2.9 Hz in control vs. 7.1 ± 2.3 Hz in DS2 (*n* = 4). The tonic conductance in thalamic relay neurons was 212 ± 47 pS (*n* = 4) in control conditions and increased to 1319 ± 263 pS in the presence of 10 μM DS2 (Figure 2B). The scatter plot in Figure 2C, illustrates the robust increase in the magnitude of the tonic conductance measured in all dLGN relay neurons with no consistent change in the decay of the IPSCs recorded from the same cells. Therefore, the increase in the tonic conductance was not associated with any significant change in the properties of sIPSCs.

### Neocortical Layer II/III pyramidal cells

**Figure 3:**
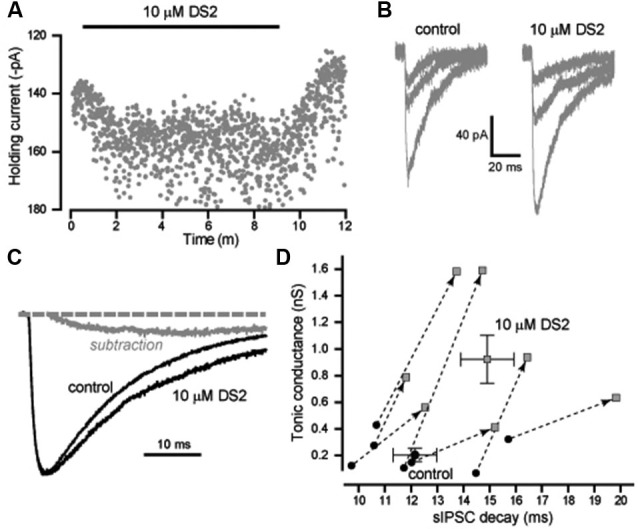
**DS2 enhances the tonic conductance and also prolongs the IPSC decay in layer II/III pyramidal neocortical neurons**. **(A)** Plot of the average holding current (V_*H*_ −70 mV) calculated every 1 s from consecutive 100 ms epochs (grey circles). The application of 10μM DS2 is indicated by the solid black bar. **(B)** Representative IPSCs detected in control conditions and during DS2 application. **(C)** Average synaptic waveform constructed from control data and in the presence of DS2. A simple subtraction of these traces has been performed to illustrate the slow rising and slow decaying DS2-sensitive component of the IPSC (grey line). The dashed line indicates the normalised baseline. **(D)** Scatter plot of the tonic conductance against the IPSC decay for each cell (grey circles) before and after DS2 application (joined by dashed line and arrow). Superimposed on this plot are the average values across all cells (mean ± SEM, *n* = 7).

We also analyzed the actions of DS2 on the phasic and tonic conductance changes in layer II/III cortical neurons. Figure 3A illustrates the enhancement of the holding current produced by DS2 application. On average, the tonic conductance was 210 ± 50 pS (*n* = 7) in control conditions increasing to 928 ± 181 pS (*n* = 7) in the presence of DS2 (*p* = 0.006). However, in contrast to other cell types, the properties of sIPSCs were significantly altered by DS2 application in these neocortical neurons. In Figure 3B, we have plotted representative examples of IPSCs that were recorded in the presence and absence of DS2. On average, the 10–90% rise-time (1.7 ± 0.1 ms vs. 1.7 ± 0.1 ms) and the peak amplitude (720 ± 46 pS vs. 677 ± 43 pS) did not alter but, the IPSC frequency significantly reduced from 8.9 ± 0.4 Hz to 7.8 ± 0.5 Hz in the presence of DS2 (*p* = 0.002) (*n* = 13). More importantly, the IPSC decay was 12.6 ± 0.6 ms (*n* = 13) in control conditions compared to 15.0 ± 0.7 ms in the presence of 10 μM DS2 (*p* < 0.001, *n* = 13, paired sample *t*-tests). This significant change in the decay of IPSCs is illustrated further in Figure 3C with the superimposition of average waveforms normalized to peak amplitude. A simple subtraction of these two waveforms illustrates how the presence of DS2 is associated with prolongation of the IPSC decay. The scatter plot in Figure 3D highlights the consistent change that occurs in both the tonic and phasic conductance following application of 10 μM DS2. In contrast to the data illustrated in Figure 2C we observe a consistent increase in both the tonic conductance and the IPSC decay recorded from all layer II/III neocortical neurons.

### DS2 actions and reduced GABA release

**Figure 4:**
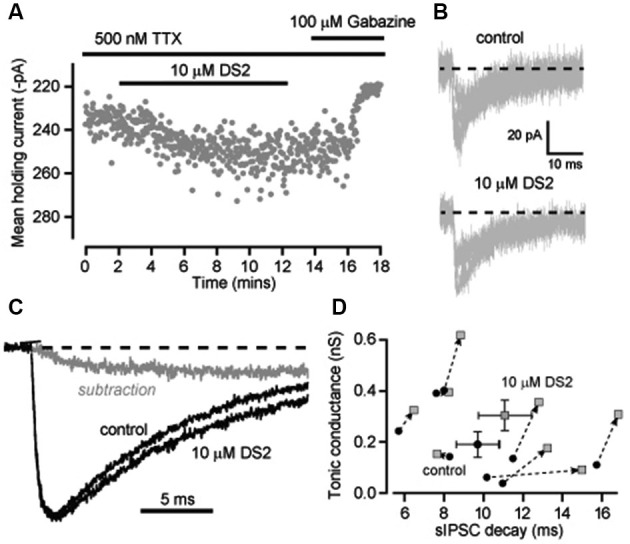
**Enhancement of the tonic conductance by DS2 in layer II/III cortical neurons is attenuated in conditions of reduced GABA release whilst still significantly increasing the IPSC decay**. **(A)** Plot of the average holding current calculated every 1 s from consecutive 100 ms epochs (grey circles) recorded from a cortical neuron during a 10 μM DS2 application followed by application of 100 μM gabazine in the continuous presence of the sodium channel blocker TTX (500 nM). **(B)** Representative mIPSCs recorded in control conditions and after DS2 application from a single cell. Dashed line indicates the normalised baseline. **(C)** Representative trace of average IPSCs recorded in the presence of TTX before and after DS2 application and the subtracted waveform (grey line). Dashed line indicates the normalized baseline. **(D)** Scatter plot of the average tonic conductance and the IPSC decay before and after DS2 application (dotted arrow) for each cell examined (grey circles) and also the average data for all cells (mean ± SEM, *n* = 8).

It is conceivable that superimposition of IPSCs is contributing to the changes in decay kinetics we have observed in recordings from neocortical neurons. In an attempt to reduce this confounding issue we blocked action potential dependent GABA release with the voltage-gated sodium channel blocker TTX and analyzed the properties of miniature IPSCs (mIPSCs) that occur at a lower frequency. The average frequency of mIPSCs was indeed reduced to 3.7 ± 0.6 Hz (*n* = 8) in the presence of TTX compared to 8.9 ± 0.4 Hz (*n* = 13) in normal recording conditions. However, the ability of DS2 to enhance the tonic conductance was much reduced in the presence of TTX. The average tonic conductance was 190 ± 49 pS in control conditions compared to 302 ± 60 pS in DS2 (*n* = 8; *p* = 0.01 paired *t*-tests). For comparison, in normal recording conditions the DS2 induced enhancement of the tonic conductance was 733 ± 186 pS (*n* = 7) but this enhancement was only 112 ± 32 pS (*n* = 8) in the presence of TTX (Figure 4A). Therefore, consistent with the view that DS2 is an allosteric modulator of δ-GABA_A_Rs, the ability of this particular drug to enhance the tonic conductance appears to be dependent upon the degree of receptor occupancy.

The ability of DS2 to prolong the decay of IPSCs was still evident in the presence of TTX. Figure 4B illustrates the superimposition of mIPSCs recorded in the presence and absence of DS2. Once again there was a small but significant change in the decay of the IPSCs. On average IPSC decay was significantly prolonged from 9.7 ± 1.1 ms in control to 11.1 ± 1.3 ms in the presence of DS2 (*n* = 8; *p* = 0.04 paired *T*-test) but all other IPSC parameters were unaltered. The IPSC frequency was 3.7 ± 0.6 Hz in control and 3.8 ± 0.6 Hz in the presence of DS2. The 10–90% rise time was 1.2 ± 0.08 ms in control and 1.3 ± 0.09 ms in DS2. The peak conductance of IPSCs was 551.1 ± 24.1 pS in control compared to 536.1 ± 19.5 pS in the presence of DS2. The prolongation of the IPSC decay is also evident in Figure 4C with the superimposition of normalized average waveforms recorded in the presence and absence of DS2 along with the subtracted waveform. The scatter plot in Figure 4D demonstrates how the prolongation of the IPSC decays was apparent in all but one cell and a small but significant increase in tonic conductance was also associated with each of these recordings.

## Discussion

### δ-GABA_A_Rs contribute differently to phasic and tonic conductance changes in cerebellum, thalamus and neocortex

This study demonstrates that DS2 induces a robust enhancement of the tonic conductance in cerebellar granule cells, dLGN thalamic relay neurons and layer II/III neocortical pyramidal cells. These cell types were specifically chosen as they are known to express high-affinity δ-GABA_A_Rs and are, therefore, capable of responding to the low resting ambient GABA concentrations present in the extracellular space to generate a tonic conductance (Brickley and Mody, 2012). In the cerebellum and thalamus the actions of DS2 are clearly restricted to the tonic conductance with no significant alterations in the properties of IPSCs. However, this was not the case for layer II/III neocortical pyramidal cells where a small but consistent slowing of the IPSC decay was apparent. DS2 prolonged the average decay of IPSCs by 20 ± 2% (*n* = 13) in normal recording conditions and by 14 ± 5% (*n* = 8) in the presence of TTX. The contribution of δ-GABA_A_Rs to IPSCs could indicate that δ-GABA_A_Rs are physically closer to GABA release sites in neocortical neurons or that those barriers to GABA diffusion may be less in the neocortex compared to the thalamus and cerebellum. We have previously suggested that receptor desensitization associated with high-affinity δ-GABARs could render these receptors unresponsive to brief changes in GABA concentration at or near the synaptic cleft (Bright et al., 2011). Our present results indicate that neocortical δ-GABA_A_Rs could be available for activation by brief changes in GABA concentration in layer II/III at least. However, more detailed information on resting GABA concentrations, δ-GABA_A_R anatomical location, and functional properties of GABA transporters are obviously required. DS2 is an allosteric modulator and its mechanism of action may well involve a reduction in the steady-state desensitization of δ-GABA_A_Rs. Assuming that drug access is also similar in the different tissues, it is also conceivable that differences in GABA affinity/efficacy are influencing DS2 action or that allosteric modulation by DS2 varies according to the δ-GABA_A_R type expressed. However, a more detailed anatomical description of δ-GABA_A_R subunit localization in the neocortex will be required before this receptor type can be considered a purely extrasynaptic receptor population in all brain regions.

The extrasynaptic location of δ-GABA_A_Rs has been firmly established for cerebellar granule cells (Nusser et al., 1998) and it is also clear that the decay of IPSCs was not altered when the δ subunit is genetically removed from granule cells (Bright et al., 2011). This would appear to contradict earlier studies that discuss the recruitment of high-affinity δ-GABA_A_Rs following GABA spillover in cerebellar granule cells (Hamann et al., 2002). This was based upon interpreting neurosteroid insensitivity of IPSCs as evidence for the involvement of the δ subunit whereas it is now clear from a number of studies that δ-GABA_A_Rs are highly sensitive to neurosteroid modulation (Stell et al., 2003; Herd et al., 2007). We have previously reported that δ-GABA_A_Rs are heavily desensitised in the presence of GABA and as such may be insensitive to GABA that has escaped from the immediate vicinity of the synapse (Bright et al., 2011). In the current study we report that DS2 has no actions on IPSC kinetics in cerebellar granule cells suggesting that synaptic release of GABA does not activate δ-GABA_A_Rs in the cerebellum. This would also appear to be the case for thalamic relay neurons of the dLGN where DS2 also only enhanced the tonic conductance. This result is consistent with previous studies using DS2 in the ventrobasal thalamus (Wafford et al., 2009) and the lack of a significant change in sIPSCs in the ventrobasal thalamus of the δ -/- mouse (Porcello et al., 2003).

The extrasynaptic location of δ-GABA_A_Rs has also been established for dentate gyrus granule cells of the mouse hippocampus (Wei et al., 2003) where δ-GABA_A_Rs have been identified at the edge of synapses in the molecular layer. At room temperature, the IPSC decay in δ -/- mice (Wei et al., 2003) was altered and the contribution of δ-GABA_A_Rs to the IPSC decay was further confirmed with another δ-GABA_A_R positive allosteric modulator (Vardya et al., 2012). This particular drug, AA29504, was reported to prolong the IPSC decay in dentate granule cells but, the tonic conductance was only enhanced in the presence of THIP. This may suggest that the extrasynaptic δ-GABA_A_Rs were not occupied by GABA in the control conditions of these experiments. AA29504 also had little action on the tonic or phasic conductance recorded in layer II/III neocortical neurons (Hoestgaard-Jensen et al., 2010). However, a slow rising and slow decaying IPSC (GABA_A_,slow) recorded from layer II/III neurons of the neocortex has been reported to involve δ-GABA_A_R activation in response to GABA released from neurogliaform cells (Szabadics et al., 2007; Olah et al., 2009). The neurosteroid THDOC, at concentrations believed to be selective for δ-GABA_A_Rs, was shown to prolong the GABA_A_,slow and enhance the tonic conductance mediated by δ-GABA_A_Rs (Szabadics et al., 2007; Olah et al., 2009). Neurogliaform axonal varicosities are not found in close opposition to classical inhibitory synapses and the GABA released from these sites is proposed to diffuse to extrasynaptic GABA_A_Rs. The IPSCs recorded in our study exhibited fast rise and decay times that are more consistent with conventional synaptic release mechanisms. Therefore, conventional fast inhibitory synaptic transmission may also involve δ-GABA_A_Rs activation in layer II/III neocortex.

### DS2 actions as a function of receptor type and receptor occupancy

As a negative control we also recorded from cerebellar Golgi cells that are situated in the internal granule cell layer and, therefore, experience a similar level of ambient GABA to cerebellar granule cells (Houston et al., 2012). Consistent with the absence of δ-GABA_A_Rs a DS2-induced conductance was absent from cerebellar Golgi cells. Initial recombinant expression data (Wafford et al., 2009) demonstrated that DS2 does not have any direct actions on δ-GABA_A_Rs but results in an enhanced maximum response to GABA with little change in GABA potency. A later study Jensen et al. (2013) suggested that this positive allosteric modulation involves DS2 binding to a novel site on the δ-GABA_A_R. This interpretation relies on the observation that DS2 enhancement still occurs in the presence of drugs that are known to bind the known orthosteric and allosteric binding sites. Additionally, loss of function mutations in the etomidate and neurosteroid binding site did not interfere with DS2 actions. Future studies using alternative techniques such as photolabelling will be required to establish if the actions of DS2 do indeed reflect binding to a previously unidentified site on the δ-GABA_A_R. Unfortunately, the therapeutic potential of DS2 is hindered by its poor brain penetration *in vivo* but, our observations on native receptors clearly demonstrate the utility of targeting the DS2 site as a strategy for enhancing tonic inhibition in brain regions that express δ-GABA_A_Rs.

Based upon *in situ* hybridization and immunohistochemical data the subunit identity of the extrasynaptic δ-GABA_A_Rs in cerebellar granule cells are of the α1/β2/β3/δ, α6/β2/β3/δ or α1/α6/β2/β3/δ type. Therefore, the ability of DS2 to enhance the tonic conductance is not greatly influenced by the α subunit identity. Indeed, the magnitude of the DS2-induced conductance was 1113 ± 366 pS (*n* = 7) in cerebellar granule cells (α6-containing), compared to 1107 ± 233 pS (*n* = 4) in dLGN thalamic relay neurons and 733 ± 186 pS (*n* = 7) in layer II/III neocortical pyramidal cells (α4-containing). However, cerebellar granule cells are considerably smaller than thalamic and cortical neurons. For example, using whole-cell measurements of cell capacitance, and assuming a specific membrane capacitance of 1 μF/cm^2^, the estimated membrane area for cerebellar granule cells is only 386 ± 30 μm^2^ (*n* = 7), compared to 10,081 ± 712 μm^2^ (*n* = 26) for thalamic relay neurons and 11,909 ± 835 μm^2^ (*n* = 29) for layer II/III neocortical pyramidal cells. The density of extrasynaptic GABA_A_ receptors is known to be fairly similar in cerebellar granule cells and neocortical pyramidal cells (Nusser et al., 1998; Kasugai et al., 2010). Therefore, it is rather surprising that cerebellar granule cells have a similar magnitude of DS2-induced conductance but this result may reflect a low level of δ-GABA_A_R occupancy (Houston et al., 2012). Indeed, in the presence of TTX the DS2-induced conductance was much reduced compared to normal recording conditions further suggesting that DS2-enhancement of the tonic conductance is dependent upon receptor occupancy. The result of this, and other studies, suggests that positive allosteric modulation of tonic inhibition via a DS2-like binding site will have distinct benefits as a strategy for enhancing tonic inhibition in the brain. In many, but not all, brain regions DS2-like binding will selectively enhance the tonic conductance and any resulting allosteric modulation will be limited to δ-GABA_A_Rs that are occupied by GABA.

## Conflict of interest statement

The authors declare that the research was conducted in the absence of any commercial or financial relationships that could be construed as a potential conflict of interest.
